# Coupling miR/isomiR and mRNA Expression Signatures Unveils New Molecular Layers of Endometrial Receptivity

**DOI:** 10.3390/life11121391

**Published:** 2021-12-11

**Authors:** Maria Nikolova, Mladen Naydenov, Ilias Glogovitis, Apostol Apostolov, Merli Saare, Nageswara Boggavarapu, Andres Salumets, Vesselin Baev, Galina Yahubyan

**Affiliations:** 1 Faculty of Biology, University of Plovdiv, Plovdiv, Bulgaria; Tzar Assen 24, 4000 Plovdiv, Bulgaria; maria.nikolova@cwh-bg.com (M.N.); mnaydenov@uni-plovdiv.bg (M.N.); ilias@uni-plovdiv.bg (I.G.); apostolbg98@gmail.com (A.A.); baev@uni-plovdiv.bg (V.B.); 2 Center for Women’s Health, 4000 Plovdiv, Bulgaria; 3 Department of Neurosurgery, Cancer Center Amsterdam, Amsterdam University Medical Centers, VU University Medical Center, De Boelelaan 1117, 1081 HV Amsterdam, The Netherlands; 4 Competence Centre on Health Technologies, 50411 Tartu, Estonia; merli.saare@ut.ee (M.S.); andres.salumets@ccht.ee (A.S.); 5 Department of Obstetrics and Gynecology, Institute of Clinical Medicine, University of Tartu, 50406 Tartu, Estonia; 6 Department of Women’s and Children’s Health, Karolinska Institute, 17177 Stockholm, Sweden; nageswara.boggavarapu@ki.se; 7 Division of Obstetrics and Gynecology, Department of Clinical Science, Intervention and Technology (CLINTEC), Karolinska Institute, 17177 Stockholm, Sweden

**Keywords:** microRNAs, isomiRs, endometrial receptivity, transcription factors

## Abstract

Embryo implantation depends on endometrial receptivity (ER). To achieve ER, the preparation of the uterine lining requires controlled priming by ovarian hormones and the expression of numerous genes in the endometrial tissue. microRNAs (miRs) have emerged as critical genetic regulators of ER in fertility and of the diseases that are associated with infertility. With the rapid development of next-generation sequencing technologies, it has become clear that miR genes can produce canonical miRs and variants—isomiRs. Here, we describe miR/isomiR expression dynamics across the four time points of natural chorionic gonadotropin (hCG)-administered cycles. Sequencing of the small RNAs (sRNA-seq) revealed that the most significant expression changes during the transition from the pre-receptive to the receptive phase occurred in the isomiR families of miR-125a, miR-125b, miR-10a, miR-10b, miR-449c, miR-92a, miR-92b, and miR-99a. Pairing the analysis of the differentially expressed (DE) miRs/isomiRs and their predicted DE mRNA targets uncovered 280 negatively correlating pairs. In the receptive endometrium, the 5′3′-isomiRs of miR-449c, which were among the most highly up-regulated isomiRs, showed a negative correlation with their target, transcription factor (TF) MYCN, which was down-regulated. Joint analysis of the miR/isomiR and TF expression identified several regulatory interactions. Based on these data, a regulatory TF-miR/isomiR gene-target circuit including let7g-5p and miR-345; the isomiR families of miR-10a, miR-10b, miR-92a, and miR-449c; and MYCN and TWIST1 was proposed to play a key role in the establishment of ER. Our work uncovers the complexity and dynamics of the endometrial isomiRs that can act cooperatively with miRs to control the functionally important genes that are critical to ER. Further studies of miR/isomiR expression patterns that are paired with those of their target mRNAs may provide a more in-depth picture of the endometrial pathologies that are associated with implantation failure.

## 1. Introduction

Millions of children have been born thanks to advances in assisted reproductive technology (ART), and although success rates have stabilized, there are still hidden endometrial factors whose dysfunction can prevent pregnancy. The disclosure of their genetic determinants would lead to an expansion of knowledge about the endometrial molecular mechanisms that determine successful implantation or its failure. Advances in next-generation sequencing (NGS) technologies have provided a new impetus to obtain a more detailed understanding of the molecular mechanisms of endometrial receptivity (ER) as well as a more detailed understanding of the dialogue between the endometrium and the embryo during the implantation process. The current knowledge of miRNAs (miRs) as molecules and their role as the key regulators of gene expression has opened up a completely new field of research and new potential clinical applications.

miRs are single-stranded molecules with a length of 19–25 nt and that are mainly encoded by their own genes—*MIR* genes, and other types of genomic loci [1,2,3]. In the canonical miR biogenesis pathway [4], the *MIR* gene is transcribed by RNA polymerase II to a primary miR (pri-miR) precursor, adopting a stem-loop secondary structure [5,6]. In the nucleus, pri-miR is cleaved by the RNase III enzyme Drosha to form a precursor of miR (pre-miR) [7,8]. Once transported to the cytoplasm, the pre-miR is further cleaved by Dicer, another RNase III enzyme, to yield a miR duplex [9]. The duplex unwinds, and the “guide strand” (mature miR) binds to the Argonaute (Ago) proteins in the miR-induced silencing complex (miRISC) [10,11]. In addition to the canonical miR biosynthetic pathway, alternative pathways that are independent of Drosha and/or Dicer have also been reported [12,13,14,15].

In addition to mature miR, a single pre-miR often generates, multiple variants that are called isoforms or isomiRs [16,17,18]. Compared to the reference sequence miR (RefSeq-miR), isomiRs may differ in length, sequence, or both [19]. Modulated or imprecise variations in cleavage by Drosha and/or Dicer generate templated isomiRs with variable 5′ and/or 3′ ends [20,21,22,23]. Non-templated nucleotide additions (NTA) that mainly involve adenylation or uridylation at miR 3′-ends by Terminal Nucleotidyl Transferases produce 3′-tailed isomiRs [24,25,26,27]. Adenosine-to-inosine modifications of pri-miRNA can interfere with pri-miR and/or pre-miR processing, leading to polymorphic isomiRs [28,29,30].

In mammals, miRs mainly act as negative post-transcriptional regulators of gene expression by directing miRISC to the target mRNA in order to mediate either mRNA degradation or translational repression [31,32]. It has been shown that isomiRs can also be functionally significant to targeting genes and pathways other than those of miRs, which significantly expands the regulatory network of *MIR* genes [29,33,34,35,36,37].

NGS technologies have made it possible to detect a vast number of isomiRs in humans [16,17,18]. A cell’s type, its condition (normal or impaired), and its development phase are the main determinants of isomiR expression [35,38]. Initially identified as the most numerous sequences that are produced by a particular *MIR* gene in a specific cell type or phase of development, the RefSeq miR may be less represented than its isomiRs in other cell types or in particular conditions [19]. Because the qualitative and quantitative characteristics of isomiRs expression profiles may vary during individual development, the isomiRs signature, in addition to gene and miR signatures, can be a promising source for developing new molecular biomarkers for biological processes, including endometrial cycle transitions and ER.

The expression profiles of endometrial miRs have been studied in natural and stimulated cycles employing high throughput techniques such as microRNA microarrays [39,40] and sRNA-seq [41,42,43,44]. A number of of DE miRs have been identified during endometrial cycle transitions and were shown to be associated with the predicted gene targets. Aberrant miR signatures have been demonstrated to be present in repeated implantation failure (RIF) patients and in infertile patients [45,46]. There are no data on the isomiRs that are involved in ER and endometrial dysfunction, an example of which is RIF. Since isomiRs can be of functional significance for ER, it is worth examining their signature in a normal fertile cycle and then transferring the acquired knowledge to female infertility-related dysfunctional ER.

Our study uses NGS to assess *MIR* and gene expression profiles at four time-points of the endometrial cycle, spanning the pre-receptive and receptive phases. We describe the dynamics of miR/isomiR expression during the phase transitions in the natural cycles that are primed by chorionic gonadotropin (hCG). We further emphasize the complexity of miR/isomiR regulation in ER by identifying DE gene targets and pathways that mediate ER. Joint analysis of the miR/isomiR and transcription factor (TF) expression identifies regulatory networks and extends our knowledge on the biological relevance of the detected isomiRs for ER.

## 2. Materials and Methods

### 2.1. Patients, Study Design, and Samples

The study protocol was approved with Ethics Committee Certificate of Approval no. 3/02.09.2019 of The Research Ethics Committee of Faculty of Biology, Plovdiv University “Paisij Hilendarski”, Plovdiv, Bulgaria. Each participant was required to provide written informed consent. 

Six healthy fertile women participated in the study. Participants fit the following selection criteria: 20–40 years of age; regularly menstruating, with a range of 21–28 days between cycles; and a BMI between 19–29 kg/m^2^. All of the participants were highly motivated volunteers. None of the participants had an accompanying disease—neither metabolic, endocrine, autoimmune, or sexually transmitted diseases, nor any gynecological infertility-associated diseases such as hydrosalpinx, endometriosis, polycystic ovarian syndrome, myomas, polyps, or any other uterine anomaly. None of the participants smoked, drank, or took any medications. All of the participants had a healthy diet. None of the participants were febrile throughout the study period. All of the participants had a history of normal pregnancy, and all of them had given birth to at least one healthy child.

Sonographic folliculometry and endometrial thickness checks (Fukuda Denshi Full Digital Ultrasound System UF-870AG, Tokyo, Japan) began on day 7, which was counted from the first day of bleeding, and these checks were repeated in the following days. Serum estradiol (E2) and luteinizing hormone (LH) levels were measured when a 17 mm follicle was observed, and following this, the same procedure was undertaken on a daily basis. Choriomon 5000 UI (IBSA Farmaceutical Italia S.r.l, Lodi, Italy was administered to control ovulation (within 36–48 h after s.c. application). It was applied subcutaneously under the following conditions: a follicle of at least 18 mm, endometrium of at least 6.5 mm, an E2 of at least 130 pmol/L, and LH less than 13 IU/l. Endometrial biopsy samples were collected from each individual on an outpatient basis and without anesthesia by Probette (Endometrial curette for microbiopsy). The biopsies were taken from the same women during their menstrual cycle, and samples were taken at four different time points that corresponded to the proliferative phase (on the day when the criteria for the application of hCG were met and before its s.c. administration) and at 2, 7, and 9 days after hCG administration. The twenty-four samples that were collected from the six participants at each time point were classified into four groups: the P group (proliferative phase), the hCG+2 group (pre-receptive phase), and the hCG+7 and hCG+9 groups, which corresponded to the two time-points of the receptive phase. The biopsies were later supplemented with RNA (Thermo Fisher Scientific) and were stored at −80 °C until use.

### 2.2. RNA Extraction and Quality Control

Total RNA isolation was performed with NucleoSpin miRNA (Macherey-Nagel) in combination with QIAGEN's phenolic reagent QIAzol, according to the manufacturer’s instructions. Approximately 50 mg of endometrial tissue samples was used for the RNA extractions for all of the time points. The quality and quantity of the RNA was checked with a Fluorometer Qubit 4 and with agarose gel electrophoresis. Purified RNA quality (RIN) and quantity were assigned by means of the Qubit RNA IQ Assay (Thermo Fisher Scientific, Waltham, MA, USA). Samples with an RNA integrity number (RIN) ≥ 7 were considered to be eligible for further analysis.

### 2.3. sRNA-Seq and Data Analysis

The sRNA-seq for the endometrial samples was performed as per the published protocols [47] and used 1 ng of the total RNA as input. The amplified libraries were purified using AMPure XP beads (1:1 ratio) (Beckman Coulter). The quantification of the purified libraries was performed using a Qubit 1X dsDNA HS assay kit (Invitrogen), and the quality of the synthesized libraries was assessed using a Bioanalyzer 2100 station with a high-sensitivity DNA assay kit (Agilent Technologies). An amount of 5 ng of purified DNA from each sample was pooled and sequenced at 1 × 100 bp using the Illumina NovaSeq Platform (National Genomics Infrastructure, SciLifeLab, Stochholm, Sweden). A quality check and adapter trimming of the FASTQ files were performed using FastQC and Trim Galore via the Galaxy platform [48]. The Galaxy in-house tool—miRGalaxy (https://github.com/Glogobyte/miRGalaxy, accessed on 1 December 2021), was used to identify and classify the isomiRs based on the read offset relative to the reference (RefSeq) miRs and the read copy number [49]. In brief, clean read mapping to a custom reference database that included the two precursor arms (5p and 3p) of RefSeq miRs (miRBase) was conducted using the Bowtie software (v1.2.0, http://bowtie-bio.sourceforge.net, accessed on 1 December 2021) [50]. After identifying the miRs and isomiRs by means of the IsoRead tool, the same module classified them into two main types—template (with subtypes) and non-template isomiRs. For differential expression analysis, the count matrices for the miRs and the isomiRs in each sample were fed to the DESeq2 (v2.11.40.6, https://bioconductor.org/packages/release/bioc/html/DESeq2.html, accessed on 1 December 2021), which was included in the miRGalaxy workflow [51]. DE miRs and isomiRs with *p* < 0.05 were used for further analysis.

### 2.4. mRNA Sequencing and Data Analysis

For mRNA sequencing (mRNA-seq), 5 ng high quality RNA (RIN ≥ 7) was used. cDNA was synthesized as previously described [52], converted to the NGS library using a Nextera XT Library Prep kit (Illumina, San Diego, CA, USA), and sequenced with a NextSeq 500 high-output 75 cycle kit (Illumina, San Diego, CA, USA). A quality check and adapter trimming of FASTQ files were performed as described for the sRNA-seq data. Clean reads were aligned to the GRCh38/hg38 human reference genome by HISAT2 [53]. Count matrices were created using the featureCounts tool according to the genome annotation and were provided to the DESeq2 package. The DE genes (DEGs) with *p* < 0.05 and |log2FC| ≥ 1 were considered for further analysis.

### 2.5. Prediction of miR/isomiR Interactions with Target Transcripts and Transcription Factors

The gene targets of the miRs and isomiRs were predicted by the miRDB database (http://mirdb.org, accessed on 1 December 2021) [54]. The MiRTar algorithm allows for custom target prediction, which was used to predict the isomiR targets. The prediction scores of the implemented algorithm were in the range of 0–100, and the candidate transcripts with target scores ≥ 50 are presented as the predicted miR targets. In our analysis, only the predicted miR/isomiR targets with target scores ≥ 80 were considered. The regulatory relationships between the miRs or isomiRs with TFs were identified by the TransmiR v2.0 database (http://www.cuilab.cn/transmir, accessed on 1 December 2021) [55] from which we only selected the literature-curated TF–miR regulation data. Although the database is intended for miRs, TF-isomiR regulations can be predicted since isomiRs and their reference miR are encoded by a common gene and are under the control of the same TFs.

### 2.6. Gene Ontology and Pathway Analysis 

Gene ontology (GO) enrichment analysis was performed using g:Profiler (version e104_eg51_p15_3922dba) with the g:SCS multiple testing correction method by applying a significance threshold of 0.05 [56]. In addition to GO, the web server includes pathways from the Kyoto Encyclopedia of Genes and Genomes (KEGG).

## 3. Results

### 3.1. sRNA-Seq and Identification of DE miRs and isomiRs During the Phase Transitions of Endometrial Cycle

A single MIR gene can generate many isomiRs with predominantly similar sequences, the collection of which represents the isomiR family of the corresponding reference miR [17]. To evaluate the expression dynamics of isomiR families during the phase transitions of the endometrial cycle, we analyzed 24 sRNA libraries from four sample groups—P, hCG+2, hCG+7, and hCG+9. Multiple isomiRs as well as multiple miRs were found to be expressed in the endometrial tissue. The identified isomiRs include template isomiRs, the ends of which are offset from one or both ends of the RefSeq miR (5-isomiRs, 3-isomiRs, and 5′3′-isomiRs), and non-template isomiRs (with 3′ additional non-template nucleotides).

**Figure 1:**
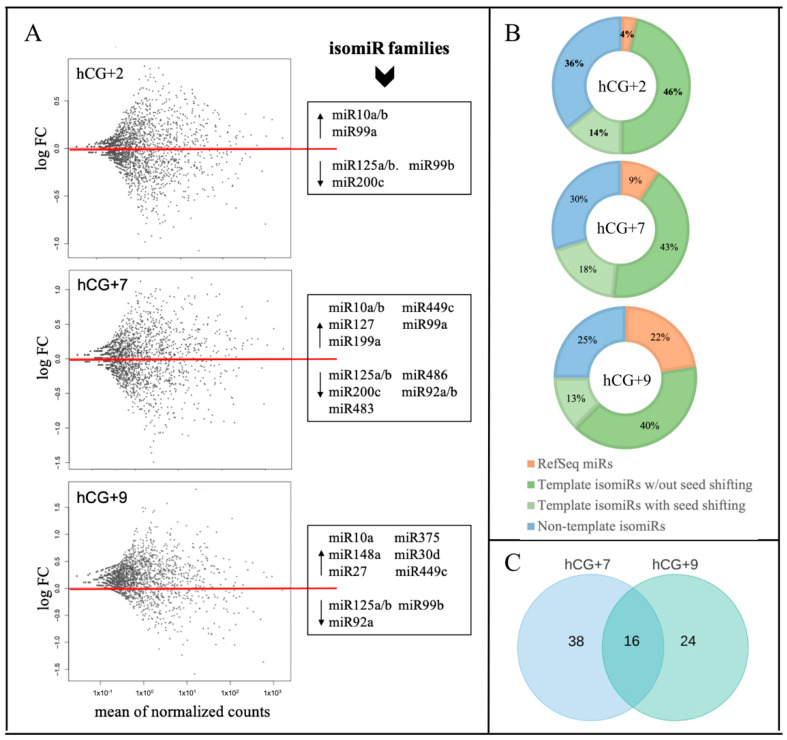
miR/isomiR dynamics during the phase transitions of the endometrial cycle. (**A**) Relative expression of miR/isomiR, where the three time points of hCG+2, hCG+7, and hCG+9 are compared to the proliferative phase; (**B**) miR/isomiR percentage distribution; (**C**) DE miR/isomiR specific to hCG+7 or hCG+9 or overlapped between hCG+7 and hCG+9.

To identify the DE miRs and the isomiRs among the analyzed time-points, the miR/isomiR expression levels at hCG+2, hCG+7, and hCG+9 were compared with those at the P phase, while the hCG+7 and hCG+9 samples represented the receptive status of the endometrial biopsies. The DE analysis showed significant changes in the miR/isomiR profiles and in the expression patterns between the transitions of the studied time-points (Figure 1A). The number of DE miRs and isomiRs as well as the absolute of their log2FC values, increased significantly during the transition from the pre-receptive (hCG+2) to receptive (hCG+7 and hCG+9) phase (Table S1). The percentage distribution of the DE miRs and isomiRs also changed during the endometrial cycle—while the template isomiRs remained the most abundant sequences for all of the time points, the percentage of RefSeq miRs increased and reached 22% for all of the sequences at hCG+9 (Figure 1B). Multiple DE isomiRs showed shifted seed regions due to the 5' end offsets relative to the reference miR (Figure 1B). The most common addition to the DE non-template isomiRs was adenine (A) (A, 2A, or 3A) (Table S1).

Members of the isomiR families of miR-125a/b, miR-92a/b, and miR-99b showed a steady downward trend in their expression levels during the transition from the pre-receptive to the receptive phase. In this transition, a reverse trend that increased the expression levels was found in the members of the isomiR families of miR-10a/b, miR-30d, miR-375, miR-449c, and miR-99a (Figure 1A). Comparing the two time points of the receptive phase, of all of the 78 DEmiRs/isomiRs (14 miRs and 64 isomiRs), only 16 were overlapped between hCG+7 and hCG+9 (Figure 1C). More significant changes in the absolute values of log2FC were found at hCG+9 compared to at hCG+7 (Table S1).

### 3.2. mRNA-Seq and Identification of DEGs during the Phase Transitions of Endometrial Cycle

**Figure 2:**
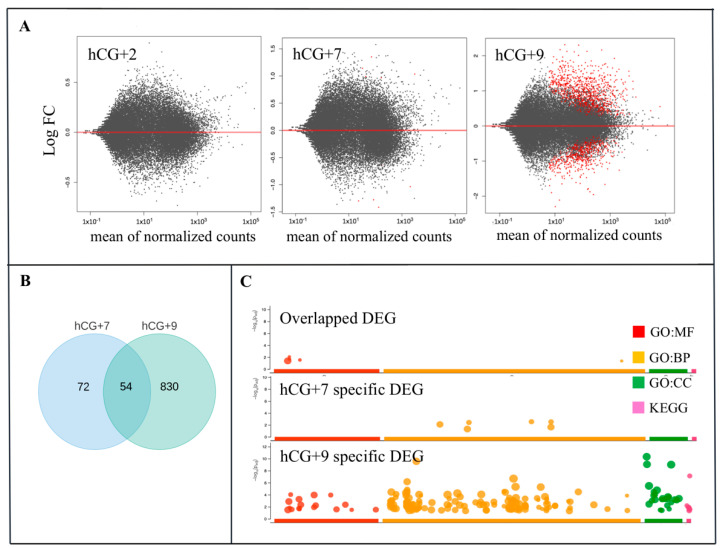
Gene dynamics during the phase transitions of the endometrial cycle. (**A**) Relative gene expression and the three time points hCG+2, hCG+7, and hCG+9 are compared with the proliferative phase; (**B**) DEGs that are specific for hCG+7 or hCG+9 or that are overlapped between hCG+7 and hCG+9. (**C**) GO ontology enrichment of the specific and overlapped DEGs. GO, gene ontology; BP, biological processes; MF, molecular function; CC, cellular component. KEGG, Kyoto Encyclopedia of Genes and Genomes.

To identify the DEGs during the phase transitions of the endometrial cycle, the gene expression levels of the hCG+2, hCG+7, and hCG+9 groups were compared to the P group. The most dramatic changes in terms of the transcriptome profiling occurred at hCG+9 compared to at hCG+2 and hCG+7 (Figure 2A). We identified 127 and 883 DEGs at hCG+7 and hCG+9, respectively (Table S2). There were 54 overlapping DEGs that were between the two time points (Figure 2B). Of all of the DEGs that were discovered at hCG+9, 614 were up-regulated, and 269 were down-regulated (Table S2).

The functional enrichment analysis of the DEGs from the two time points of the receptive phase provided an overview of their functional roles in ER (Figure 2C). The largest number of enriched GO terms were found within the DEGs at hCG+9. At this time point, the GO molecular function (GO:MF) category was enriched in several ways, including chemokine activity, transmembrane transporter activity, chemokine receptor binding activity, receptor ligand activity, among others. The GO:BP (biological processes) category included terms such as response to external stimulus, defence response, localization, regulation of biological quality, etc. According to KEGG analysis, the DEGs were associated with pathways such as mineral absorption, complement and coagulation cascades, among others. The GO analysis of the hCG+7-specific DEGs identified molecular functions and biological pathways that have been shown to be associated with cytokine and type I interferon signalling. 

### 3.3. Interactions of DE miRs and isomiRs with DEGs in the Receptive Endometrium

MiR target prediction is critical for understanding miR functions. The gene targets of the DE miRs and isomiRs from the endometrial cycle phases were predicted by employing the miRDB database (Table S3). IsomiR target search was performed by applying the custom prediction interface. Any 3′-isomiRs with the same seed region as the corresponding miRs shared the same target genes. In contrast, the 5′-isomiRs, which tend to have an altered seed region relative to miRs, were associated with a different set of target genes. The top 10 targets of the DE 5′-isomiRs during the transition from the pre-receptive to the receptive phase and of their corresponding miRs are presented in Table S3. A comparison of the target scores of the 5′-isomiRs with those of the miRs show that the targets of some of the 5′-isomiRs may have higher target score values than those of the reference counterparts (Table S3). These are the 5′- and 5′3′ isomiRs of miR-449c-5p, which have 268 predicted targets and an average target score that is much higher than that of the 142 predicted targets of the reference miR. Another 5′-isomiR, which is also characterized by high target score values, is that of miR-30d-5p.

**Figure 3:**
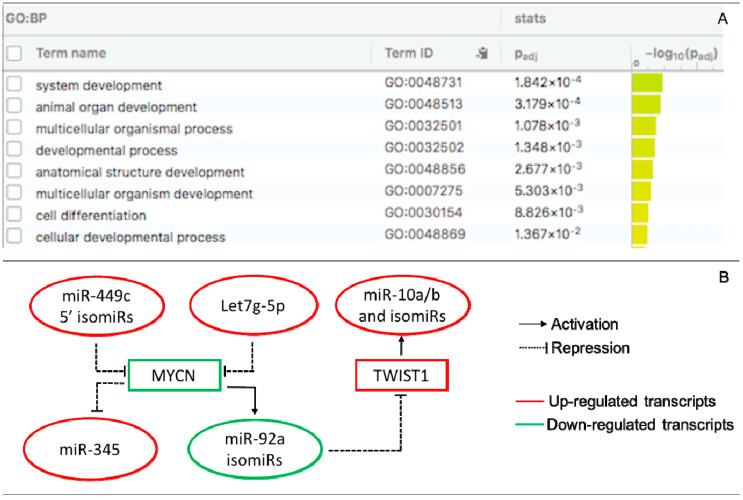
Interactions of DE miRs and isomiRs with DE target transcripts and TF in the receptive endometrium. (**A**) GO ontology enrichment of DE target transcripts. GO:BF are presented; (**B**) TF-miR/isomiR-gene regulatory circuit. MYCN is a target of miR-449c 5′-isomiRs and let7g-5p and regulates the expression of miR-92a isomiRs and miR-345. TWIST1 is a target of miR-92a isomiRs and regulates the expression of the miR-10a and miR-10b isomiR families.

Pairing analysis between the predicted targets of the DE miRs/isomiRs and DEGs (mRNAs) uncovered 280 negative correlating pairs and 281 positive correlating pairs in the receptive endometrium (Table S3). Since miRs mainly act as negative post-transcriptional regulators of their transcript targets, we present the negatively correlated miRs/isomiRs and their targets in receptive endometrium in Table 1. Among them, we identified eighteen transcripts that were targets of more than one miR or isomiR. MYCN, FGF7, and CNTN1 were the most altered targets in the receptive endometrium compared to in the pre-receptive endometrium, of which MYCN had the highest target score (for its targets – the 5′3′-isomiRs of miR-449c). Functional enrichment analysis showed the enrichment of several GO biological process (GO:BP) terms, including system development, animal organ development, multicellular organismal process, and developmental process (Figure 3A).

Transcription factors are critical regulators of developmental and tissue-specific gene expression, including *MIR* genes. To gain information on the TF-miR regulations that take place during the endometrial cycle, we identified any TFs that may regulate DE miRs/isomiRs at the receptive phase by searching the TransmiR 2.0 database. The literature-curated TF-miR relationships were associated with DE miRs/isomiRs (Table S4). We found two DE isomiR families (miR-10a/b and miR-92a) and two DE miRs (miR-31-5p and miR-345) that were linked with three DE TF (MYCN, TWIST1, and CEBPB) upon the transition from the pre-receptive to the receptive phase. Based on these data, a regulatory TF-miR/isomiR-gene target circuit that includes let7g-5p; miR-345; the isomiR families of miR10a/b, miR-92a, and miR-449c; and MYCN and TWIST1 was proposed to take place during ER establishment (Figure 3B).

## 4. Discussion

High-throughput sequencing has revealed that *MIR* genes give rise to multiple isomiRs in a tissue-specific manner that can be determined by an organism’s specific physiological or pathological conditions. Unlike most isomiRs, which are functionally redundant compared to their reference counterparts, 5′-isomiRs have different target spectra due to a shifted seed sequence. All of this suggests that isomiRs have a functional significance, which may provide an additional layer of gene expression regulation. In addition, it is believed that some isomiRs may be useful as diagnostic and prognostic markers of various diseases, with applications in patient care and therapy.

Several studies have been reported in the literature that provide a qualitative and quantitative assessment of healthy endometrial miRNome [41,42,43,44]. Our study was justified by the existing gap in our knowledge of isomiR expression dynamics in the endometrial cycle and the likely involve of isomiR l in the regulatory networks underlying the ER. In order to describe isomiR profiles throughout the endometrial cycle, we applied sRNA-seq technology to measure small RNA expression levels in the endometrial biopsies that were collected at different four time points of the same endometrial cycle. Unlike the studies mentioned above, hCG was administrated to achieve ovulation within 36–48 h and to achieve a more accurate dating of the receptive phase. In a previous study that evaluated the effect of hCG on ER in parallel with natural ovulation cycles, no significant differences were found [57]. In our study, the greatest variations in miR/isomiR expression were observed at hCG+9, which correlated with significant changes in the gene expression levels. We identified 14 DE miRs in the receptive endometrium of the hCG-administered natural cycles, as some of them were consistent with those that had been determined in spontaneous ovulatory cycles [41,42,43,44] and during hCG-stimulated cycles during IVF treatment [41].

To the best of our knowledge, this work is the first effort to profile the isomiR changes that are associated with healthy human endometrium phase transitions. The sRNA-seq data analysis revealed that several isomiR families changed across the studied time-points within the cohort of patients. The isomiR families that showed the largest number of members with altered expression were those of miR125a/b, miR10a/b, miR449c, miR92a/b, and miR99a.

The isomiR families of miR-125a, miR-125b, miR-92a, and miR-92b showed decreased expression throughout the pre-receptive and receptive phases compared to during the proliferative phase. Among their DE variants, 3′-isomiRs were predominant, and some of them had an additional A or U. Previous studies have implicated miR-125a in metabolic and pro-angiogenic profiling to control vascular morphology and function in inflammatory diseases and cancers [58,59], and its deficiency has been shown to be related to enhanced angiogenic processes through the metabolic reprogramming of the endothelial cells [60]. The other family member, mir-125b, influences the proliferation and apoptosis of tumor cells [61,62]. Both miR-92a and miR-92b belong to the miR-17-92a cluster. It has been shown that miR-92a inhibition accelerates angiogenesis and blood circulation in ischemic tissues [63] and enhances fracture healing by promoting angiogenesis in mice [64]. The endometrium exhibits regular non-pathological angiogenesis when restoring the vascular bed [65]. The isomiR families of miR-125a, miR-125b, miR-92a, and miR-92b could contribute to the angiogenic processes that take place in the dynamic endometrial vasculature, which grows and remodels itself during each menstrual cycle. Here, we identified several 3′-isomiRs belonging to miR-10a and miR-10b, which were up-regulated during the pre-receptive and receptive phases. Their reference counterparts are expressed at different levels in ovarian cells, such as in the granulosa, theca, and stroma cells, and have been reported to participate in fine-tuning cell proliferation and apoptosis in the female reproductive system [66].

In our study, the isomiR family of miR-449c was one of the most strongly up-regulated families during the transition from the pre-receptive to receptive phase. The members of the miR449 family (miR-449a/b/c) were discovered among the most highly upregulated miRs 7–9 days after ovulation [42,43]. Here, we identified several distinct DE 5′3′- and 3′-variants of miR-449c while the reference miR showed no significant expression changes during the transition. miR-449c is a part of the miR-34/449 superfamily, which comprises six homologous miRs (miR-34a, miR-34b/c, and miR-449a/b/c). Of them, miR-449c and miR-34b differ from the other family members based on their seed region (as referenced in miRbase). The ER-related 5′-isomiRs of miR-449c that we discovered share the same seed region with miR-34a/c and miR-449a/b. Mercey and co-workers [37] described two 5′-isomiRs of miR-34/449 that had the same seed region as the ER 5′-isomiRs and that displayed functional differences in human airway epithelial cells than they did in the reference counterparts. It was reported that miR-449a/b had a much more potent inhibitory effect on cell proliferation and migration in cancer compared to miR-449c [67]. Because the ER 5′-isomiRs of miR-449c share the same target repertoire as miR-34a/c and miR-449a/b, we can assume that these isomiRs may be involved in the control of the different proliferative activities of the cells that are in the endometrium.

Among the DEGs in the receptive endometrium, our attention was drawn to *MYCN*. It was predicted to be a target of the 5′-isomiRs of miR-449c and let7g-5p, as it demonstrated a very high target score (98). We observed a negative correlation between the expression levels of MYCN mRNA (down-regulated) and those isomiRs (up-regulated) in the receptive phase, suggesting the functional significance of the miR-449c isomiR family as a negative regulator of MYCN. The gene is a member of the MYC family and functions by up- and down-regulating genes directly by promoter binding or indirectly via mediators [68]. MYCN was reported to activate miR-92a and to repress miR-345 [69,70,71]. In line with the observed MYCN-miR-92a and MYCN-miR-345 correlations, the under-representation of the MYCN transcript was associated with the down-regulation of the miR-92a isomiRs and the up-regulation of miR-345 in the receptive endometrium. Our data also suggest that, in turn, reduced levels of miR-92a isoforms may lead to the increased expression of its predicted target TWIST1. A number of TWIST1 studies have shown the complex role of this TF in normal and disease states [72]—it plays a significant role in organ development, including in the initiation of uterine decidualization [73], but it may also promote cancer metastasis. Thus, the regulatory network that was described here requires further study to confirm its role in the molecular mechanisms underlying the ER.

The small number of patients was the main limitation of our study, which is common for assays requiring invasive sampling [39,43,74,75]. In order to minimize inter- and intra-patient variability, endometrial biopsies were taken from the same individuals at four time points that could potentially affect gene expression at each subsequent point due to the local damage caused by the previous biopsy. Therefore, an important next step is the validation of the in silico predicted miRs/isomiRs and their target transcripts by RT-qPCR in a larger number of individuals throughout normal and/or dysregulated menstrual cycles.

## 5. Conclusions

Our work uncovers the complexity and dynamics of the endometrial isomiRs that can act cooperatively with miRs to control functionally related genes in the establishment of ER. Further studies of miR/isomiR expression patterns paired with those of their target mRNAs may provide a more in-depth picture of menstrual cycle disorders in women suffering from infertility and RIF. Therefore, it is worth investigating the possibility of miRs/isomiRs being used as biomarkers for endometrial receptivity.

## Figures and Tables

**Table 1:** Negatively correlating DE miRs/isomiRs and gene targets in the receptive endometrium. miRs/isomiRs are classified in two groups—RefSeq miR and/or 3′isomiRs sharing the same seed region (A), and 5′isomiRs (B). For each group, the representative with the expression that changed the most is provided. Seed region is underlined. ↑ up-regulated, ↓ down-regulated.

DE miRs/isomiRs				DE Targets *	
Family Name	miR/isomiR Name		miR/isomiR Sequence		
let-7g-5p ↑	A	let-7g-5p	CUGUACAGGCCACUGCCUUGC	↓	MYCN^2^, KCNC2, FAXC
miR-10a/b ↑	A	miR-10b-5p	UACCCUGUAGAACCGAAUUUGUG	↓	GABRB2
	B	miR-10a-5p_t_+1_-1	ACCCUGUAGAUCCGAAUUUGU	↓	CPED1
miR-23a ↑	A	miR-23a -3p_t_0_-1	AUCACAUUGCCAGGGAUUUC	↓	FUT9^4^, ROBO2^3^, CRISPLD1, FREM1, ANO4^2^, FBN2, KCNIP4^2^, GUCY1A2, SH3BGR, ZNF730
miR-27b ↑	A	miR-27b-3p	UUCACAGUGGCUAAGUUCUGC	↓	EYA4^3^, SFRP1, RARA^2^, BMP3^3^, CCN4^2^, SLC9A4, CYP39A1^2^
	B	miR-27b-3p_t_+1_0	UCACAGUGGCUAAGUUCUGC	↓	CYP39A1^2^, LBH^2^, EYA4^3^, RARA^2^, CCN4^2^
miR-30d ↑	A	miR-30d-5p_t_0_+2	UGUAAACAUCCCCGACUGGAAG	↓	RTKN2^2^, ANO4^2^, SPOCK3^2^, RAPGEF4, GRIA2, OVOL1, PAPOLB, GCLC^2^, CAMK4, NAP1L2, SEMA3A
	B	miR-30d-5p_t_+1_+2	GUAAACAUCCCCGACUGGAAGCU	↓	RTKN2^2^, ROBO2^3^, GCLC^2^, NDNF, ANG, SPOCK3^2^, IFIT, FHOD3, MYH15, CSMD3, FUT9^4^, ESCO2
miR-31 ↑	A	miR-31-5p	AGGCAAGAUGCUGGCAUAGCU	↓	LBH^2^
miR-92a ↓	A	miR-92a-3p_nont_0_+2_AA	UAUUGCACUUGUCCCGGCCUGUAA	↑	GRHL1, TTC9, MTF1^2^, IDH1, SOX11, MMP10, REXO1, IRS2, TWIST1, LRRC1, PIK3AP1
miR-125a/b ↓	A	miR-125a-5p_t_0_-2	UCCCUGAGACCCUUUAACCUGU	↑	CYP24A1, MTF1^2^, SLC7A1, ANKRD33B, PHACTR3, TMPRSS13, SOD2, IGSF11
	B	miR-125a-5p_t_+1_-2	CCCUGAGACCCUUUAACCUGU	↑	TRNP1, DPP4, STAC2, CPT1A, ADCY1, ADAMTS8
miR-127 ↑	A	miR-127-3p_nont_0_+2_AU	UCGGAUCCGUCUGAGCUUGGCUAU	↓	ATP1A2
miR-141 ↑	A	miR-141-3p	UAACACUGUCUGGUAAAGAUGG	↓	ELMOD1^3^, NAP1L2, CNTN1, HCN1, MCIDAS^2^, TMEM130
miR-148a ↑	A	miR-148a-3p_t_0_+1	UCAGUGCACUACAGAACUUUGUC	↓	ADGRB3, ROBO2^3^, BMP3^3^, ISM1
miR-199a ↑	A	miR-199a-5p	CCCAGUGUUCAGACUACCUGUUC	↓	HMCN1
	B	miR-199a-5p_t_+1_0	CCAGUGUUCAGACUACCUGUUC	↓	PBK, EYA4^3^, KCNIP4
miR-200c ↓	A	miR-200c-3p_t_0_-3	UAAUACUGCCGGGUAAUGAU	↑	PPP1R9B, NTRK2, KYNU, PITPNM3, PROK2, CDYL2, KLF6, SLC39A14, ADH1B,
miR-449c ↑	A	miR-449c-5p_t_0_-2	UAGGCAGUGUAUUGCUAGCGGCU	↓	ELMOD1^3^, NEXMIF, FUT9^4^
	B	miR-449c-5p_t_+1_-1	AGGCAGUGUAUUGCUAGCGGCUG	↓	MYCN^2^. FUT9^4^, ELMOD1^3^, BMP3^3^, ASIC2, NCEH1, MCIDAS^2^,
miR-486 ↓	A	miR-486-5p	UCCUGUACUGAGCUGCCCCGAG	↑	FGF7, CEMIP

* Uppercase indicates the number of miRs and/or isomiRs presented in the table that are predicted to target the specified gene.

## Data Availability

Data are contained within the article or in the supplementary material.

## Supplementary Materials

The following are available online at https://www.mdpi.com/article/10.3390/life11121391/s1, Table S1: DE miRs/isomiRs at hCG+2, hCG+7, and hCG+9 vs. proliferative phase, Table S2: DEGs at hCG+2, hCG+7, and hCG+9 vs. proliferative phase, Table S3: DE targets of DE miRs/isomiRs in the receptive endometrium, Table S4: Literature-curated TF of DE miRs/isomiRs in the receptive endometrium.
